# Effects of cooling on muscle function and duration of stance phase during gait

**DOI:** 10.1186/2046-7648-4-S1-A47

**Published:** 2015-09-14

**Authors:** Amitava Halder, Chuansi Gao, Michael Miller

**Affiliations:** 1 Thermal Environment Laboratory, Division of Ergonomics and Aerosol Technology, Department of Design Sciences, Faculty of Engineering, Lund University, Lund, Sweden; 2 Division of Physiotherapy, Department of Health Sciences, Faculty of Medicine, Lund University, Lund, Sweden

## Introduction

Cold exposure alters muscular function. Muscle cooling influences the neuromuscular activation during maximal isometric voluntary contractions (MVC) and the amplitude of surface electromyography (sEMG) [1,2]. It also slows down the mechanical process during contraction [3]. The purpose of this study was to investigate the effects of local cooling in cold water at 10 °C for 20 min in a climate chamber on lower leg muscle activity and gait pattern.

## Methods

Sixteen healthy adults (eight females), with a mean age of (SD) 27.0 (2.9) years; body mass 66.3 (9.8) kg; and height 169.5 (7.8) cm participated in this experimental study. The median frequency (MF) and mean power frequency (MPF) of sEMG from tibialis anterior (TA) and gastrocnemius medialis (GM) muscles during MVC in ankle planter (PF) and dorsi-flexion (DF) against a hand-held dynamometer as well as contact times on a force plate during gait before and after cooling were measured and analysed.

## Results

The MF and MPF were significantly lower (P < 0.01*) in both TA and GM muscle during MVC and in TA during gait trials after cooling. However, the frequency analysis for GM muscle showed no significant difference (p = 0.46 and 0.06, respectively) either in MF or MPF during walking on level surface (table 1).

**Table 1:** The means and SD (Hz) for the MF and MPF of the TA and GM during gait and MVC trials before and after cooling (N = 16).

sEMG	Tibialis Anterior (TA)		Gastrocnemius Medialis GM	
	**Pre Cooling**	**Post Cooling**	**Pre Cooling**	**Post Cooling**

**Gait MF**	83.0 ± 10.2*	69.9 ± 9.6*	81.6 ± 12.6	79.3 ± 11.1

**Gait MPF**	99.7 ± 11.5*	82.3 ± 11.7*	99.8 ± 13.2	93.2 ± 12.4

**MVC MF**	87.0 ± 9.7*	50.0 ± 6.1*	111.7 ± 16.7*	90.8 ± 14.8*

**MVC MPF**	100.7 ± 10.6*	59.8 ± 7.7*	129.1 ± 15.3*	101.0 ± 16.1*

Additionally, the post-cooling stance phase over the force plate was longer than pre-cooling (p = 0.013).

## Discussion

The significant time difference might be caused by the cold induced MF and MPF decrease in sEMG. Our previous investigation reported that cooling increased the sEMG amplitude and produced fatigue like responses in the leg muscles [2]. Moreover, other studies showed that muscle fatigue resulted in electromechanical delay during cold exposure [1,4].

## Conclusion

Moderate degree and duration of cooling may affect muscle motor unit firing rates, thus shifting the sEMG spectrum to lower frequencies, therefore decreasing the leg muscle force production. The result suggests that muscle cooling can cause cold induced frequency decrease in sEMG similar to fatigue response and lead to reduced muscle performance.

**Figure 1:**
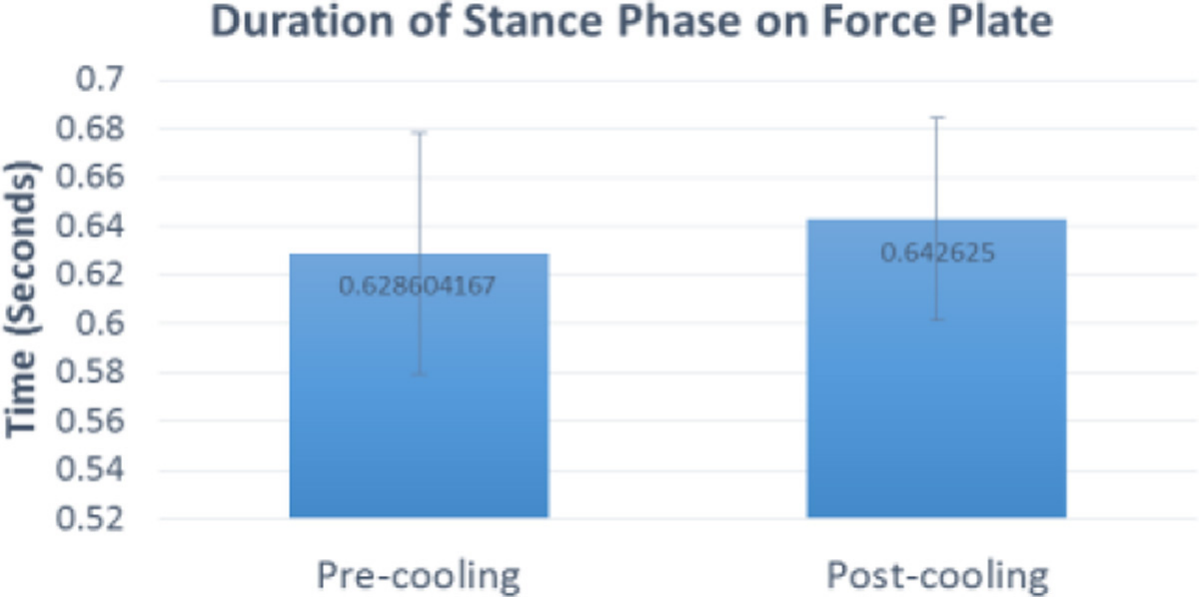
**Duration of stance phase in gait trials**.
